# Neuronal TRPV1 activation regulates alveolar bone resorption by suppressing osteoclastogenesis via CGRP

**DOI:** 10.1038/srep29294

**Published:** 2016-07-08

**Authors:** Naoki Takahashi, Yumi Matsuda, Keisuke Sato, Petrus R. de Jong, Samuel Bertin, Koichi Tabeta, Kazuhisa Yamazaki

**Affiliations:** 1 Laboratory of Periodontology and Immunology, Division of Oral Science for Health Promotion, Niigata University Graduate School of Medical and Dental Sciences, Niigata, Japan; 2 Division of Periodontology, Department of Oral Biological Science, Niigata University Faculty of Dentistry, Niigata, Japan; 3 Research Center for Advanced Oral Science, Niigata University Graduate School of Medical and Dental Sciences, Niigata, Japan; 4 Sanford Burnham Prebys Medical Discovery Institute, NCI-Designated Cancer Center, La Jolla, CA, USA; 5 Department of Medicine, University of California, San Diego, La Jolla, CA, USA

## Abstract

The transient receptor potential vanilloid 1 (TRPV1) channel is abundantly expressed in peripheral sensory neurons where it acts as an important polymodal cellular sensor for heat, acidic pH, capsaicin, and other noxious stimuli. The oral cavity is densely innervated by afferent sensory neurons and is a highly specialized organ that protects against infections as well as physical, chemical, and thermal stresses in its capacity as the first part of the digestive system. While the function of TRPV1 in sensory neurons has been intensively studied in other organs, its physiological role in periodontal tissues is unclear. In this study we found that *Trpv1*^−/−^ mice developed severe bone loss in an experimental model of periodontitis. Chemical ablation of TRPV1-expressing sensory neurons recapitulated the phenotype of *Trpv1*^−/−^ mice, suggesting a functional link between neuronal TRPV1 signaling and periodontal bone loss. TRPV1 activation in gingival nerves induced production of the neuropeptide, calcitonin gene-related peptide (CGRP), and CGRP treatment inhibited osteoclastogenesis *in vitro*. Oral administration of the TRPV1 agonist, capsaicin, suppressed ligature-induced bone loss in mice with fewer tartrate-resistant acid phosphatase (TRAP)-positive cells in alveolar bone. These results suggest that neuronal TRPV1 signaling in periodontal tissue is crucial for the regulation of osteoclastogenesis via the neuropeptide CGRP.

The transient receptor potential (TRP) superfamily is one of the largest ion channel families, and is thought to play a pivotal role in sensing temperature, pain, and noxious substances^1^. TRP channels are found in all animals – from flies to humans – and are highly conserved among species^2^. Twenty-eight TRP channels have been described in mammals, which are divided into six subgroups based on similarity in amino acid sequences: TRP cation channel subfamily C (canonical; TRPC), subfamily V (vanilloid; TRPV), subfamily M (melastatin; TRPM), subfamily A (ankyrin; TRPA), subfamily P (polycystic; TRPP), and subfamily ML (mucolipin; TRPML). The channels have six transmembrane spanning domains with a pore-forming loop between the fifth and sixth segments^3^. Consistent with the diversity in intracellular amino and carboxyl termini, TRP channels have diverse modes of activation, ion selectively, and physiological functions^4^. TRPV1, the founding member of the TRPV subfamily, is a non-selective cation channel activated by noxious heat, protons, and vanilloids such as capsaicin, the pungent ingredient in chili pepper^5^. TRPV1 is abundantly expressed in peripheral sensory nerves and acts as a nociceptor (i.e., pain receptor)^6^,^7^,^8^. In addition to this prototypical function of TRPV1, there are several reports that implicate this ion channel in the pathogenesis of diseases such as bladder disorders^9^, colitis^10^, and cancer^11^. Furthermore, recent studies have demonstrated the pivotal role of TRPV1 in the pathogenesis of osteoporosis^12^ and osteoarthritis^13^ in experimental models by genetic and/or pharmacological manipulation of the channel. However, the contribution of TRPV1 in these bone-related diseases remains controversial due to multiple conflicting reports^12^,^13^.

CGRP is a neuropeptide that is widely distributed in the peripheral and central nervous systems^14^. It has been previously demonstrated that the local administration of capsaicin induces the release of CGRP from sensory nerve endings^15^, and that TRPV1 antagonists prevent this effect^16^. It is well documented that the local release of CGRP from sensory nerve endings can modulate vasodilation^17^, inflammatory responses^18^, and osteogenesis^19^. *In vitro* and in a number of experimental animal models, CGRP has been shown to inhibit osteoclast activity and bone resorption^19^. These findings suggest that nerve fibers, via the release of CGRP, could regulate bone remodeling^20^.

The oral cavity, a gate-keeper in the alimentary canal, is particularly exposed to external damage, including traumatic challenges, changes in pH and temperature, and a variety of noxious compounds. The sensory receptor TRPV1 is abundantly expressed in the dental pulp^21^, salivary glands^22^, tongue and gingiva^23^. As the nervous system contributes to the pathophysiology of various diseases, it is now evident that neuropeptides may also be involved in the initiation and progression of oral diseases^24^. Periodontitis, one of the major inflammatory oral diseases, is characterized by inflammation of the gingiva and destruction of alveolar bone^25^. The identification of CGRP in both gingival tissue and gingival crevicular fluid (GCF) has suggested its pathological implications in periodontitis^26^,^27^. Despite the fact that one report demonstrated co-localization of TRPV1 and CGRP in peripheral nerve fibers in gingival tissues^28^, a functional link between neuronal TRPV1 signaling and CGRP in the context of periodontitis has not been established yet. In this study we addressed the role of the neuronal TRPV1-CGRP signaling axis in the pathogenesis of periodontitis.

## Results

### TRPV1 deficiency exacerbates experimental murine periodontitis

**Figure 1:**
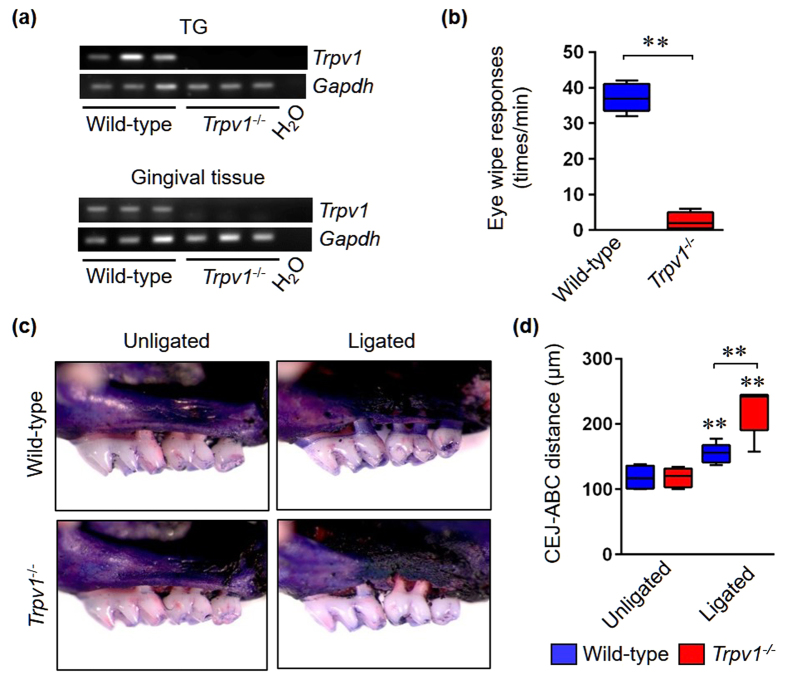
Genetic deletion of *Trpv1* increases alveolar bone loss in the ligature-induced periodontitis model. (**a**) Validation of *Trpv1* deletion by RT-PCR. Expression of *Trpv1* transcripts was tested in three biological replicates of TG and gingival tissue from wild-type and *Trpv1*^−/−^ mice, respectively. *Gapdh* was used as an internal control. H_2_O samples were used as a negative control. (**b**) Validation of ablation of functional TRPV1 by eye-wiping test (n = 6 in each group). All data are mean ± SD (**p < 0.01 as indicated, by Mann-Whitney U-test). (**c**) Representative stereoscope images of defleshed maxilla from wild-type and *Trpv1*^−/−^ mice in unligated and ligated groups at day 7. (**d**) Quantification of alveolar bone loss measured by the distance from CEJ to ABC (n = 6 in each group). All data are mean ± SD (**p < 0.01 versus unligated wild-type or as indicated, by ANOVA).

First, we validated the deletion of *Trpv1* transcripts in trigeminal ganglia (TG) and gingival tissue in *Trpv1*^−/−^ mice by RT-PCR (Fig. 1a). We confirmed functional deficiency of TRPV1 by the ocular administration of capsaicin, i.e. the eye-wiping test (Fig. 1b). To investigate the possible pathophysiological role of TRPV1 in periodontal diseases, we used the ligature-induced periodontitis model^29^ (Supplementary Fig. S1a,b). Interestingly, the degree of bone destruction in ligated *Trpv1*^−/−^ mice was significantly greater when compared with ligated wild*-*type mice (Fig. 1c). The level of alveolar bone crest was similar in *Trpv1*^−/−^ mice and wild-type mice in unligated groups, indicating that there were no abnormalities in skeletal development and bone phenotype in absence of *Trpv1* gene expression (Fig. 1c). Quantitative analysis was used to measure the distance between the cementoenamel junction (CEJ) and alveolar bone crest (ABC) of mesial root in the second upper molars (Supplementary Fig. S1c). CEJ-ABC distance was significantly greater in ligated *Trpv1*^−/−^ mice compared with ligated wild-type mice (Fig. 1d), suggesting that genetic deletion of TRPV1 exacerbated experimental periodontal disease in this murine model.

### Chemical ablation of neuronal TRPV1 mimics the phenotype of *Trpv1* ^−/−^ mice

**Figure 2:**
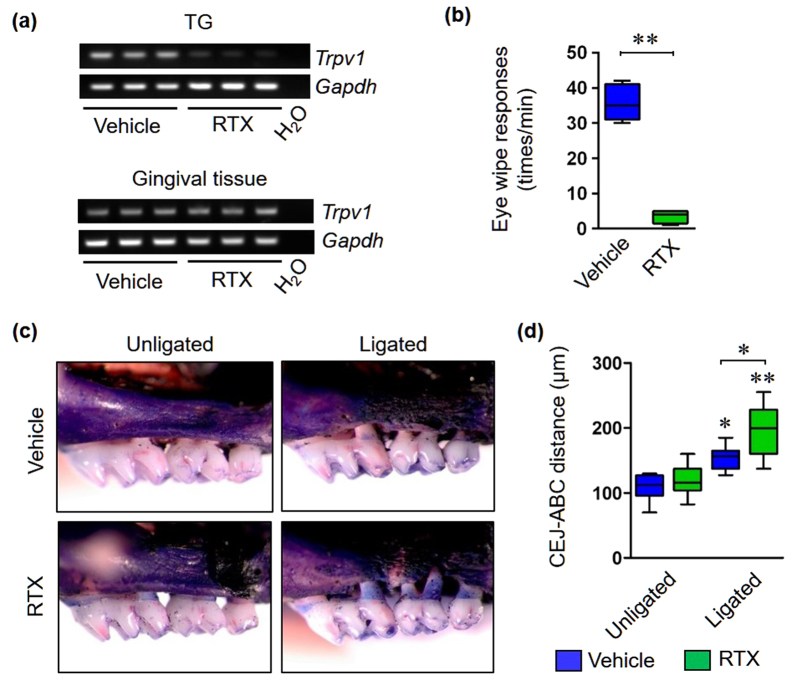
Neonatal ablation of neuronal TRPV1 exacerbates bone resorption. (**a**) Validation of neuronal deletion of *Trpv1* by RT-PCR. Expression of *Trpv1* transcripts was tested in three biological replicates of TG and gingival tissue from vehicle- and RTX-treated mice, respectively. *Gapdh* was used as an internal control. H_*2*_O samples were used as a negative control. (**b**) Validation of TRPV1^*+*^ neuronal ablation by the eye-wipe test (n = 6 in each group). All data are mean ± SD (**p < 0.01 as indicated, by Mann-Whitney U-test). (**c**) Representative stereoscope photos of defleshed maxilla from RTX-treated mice and vehicle-treated mice in unligated and ligated groups, respectively. (**d**) Quantification of alveolar bone loss was performed through measurements of CEJ-ABC distance (n = 6 in each group). All data are mean ± SD (*p < 0.05 and **p < 0.01 versus unligated vehicle-treated mice or as indicated, by ANOVA).

We have previously reported that TRPV1 is also expressed by non-neuronal cells in periodontal tissues such as gingival epithelial cells and fibroblasts^30^. To characterize the function of neuronal TRPV1, we generated mice in which TRPV1 expressing (TRPV1^+^) neurons were ablated by neonatal administration of the ultrapotent TRPV1 agonist, resiniferatoxin (RTX)^31^. Successful RTX-mediated ablation of TRPV1^+^ neurons was confirmed by PCR and eye-wiping tests (Fig. 2a,b). Ligated RTX-treated mice showed more severe bone destruction compared with ligated vehicle-treated mice (Fig. 2c). Quantitative analysis indicated that bone loss was significantly greater in ligated RTX-treated mice compared with ligated vehicle-treated mice (Fig. 2d). Taken together, these results suggest that neuronal TRPV1 signaling plays an important role in the protection against alveolar bone destruction.

### Innervating nerves in gingiva release CGRP upon TRPV1 activation

**Figure 3:**
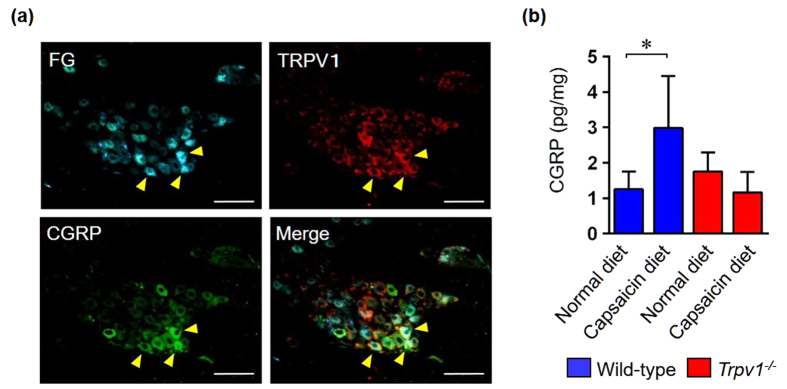
TRPV1 activation and CGRP release by nerves innervating the gingiva. (**a**) Retrograde labelled Fluoro-gold™ (FG) and immunofluorescence staining of TRPV1 and CGRP in TG. Images of trigeminal neurons stained for FG (upper left panel), TRPV1 (upper right panel), CGRP (lower left panel), and an overlay of all three staining shown in the lower right panel. Arrows indicate FG-labelled neurons that co-express TRPV1 and CGRP. Scale bars represent 100 μm. (**b**) Quantification of CGRP release from TG after oral administration of capsaicin for 7 days. The dissected TG were weighed and then incubated for 24 h at 37 °C in culture medium. CGRP protein levels in the supernatant were quantified by ELISA (n = 6 in each group). All data are mean ± SD (*p < 0.05 as indicated, by ANOVA).

Afferent sensory nerve fibers in periodontal tissues originate in the TG^32^,^33^. CGRP is one of the most prominent neuropeptides that is synthesized in neuronal cell bodies in the TG, transported anterogradely, and subsequently released in the gingiva in response to peripheral stimuli. Since the nervous system contributes to the pathophysiology of many peripheral inflammatory diseases, we hypothesized that TRPV1-mediated CGRP release in gingival tissues could play an important role in our model. First, we examined whether nerve fibers in gingival tissue that project from the TG co-express TRPV1 and CGRP by immunofluorescencestaining and fluoro-gold™ (FG) retrograde labeling. We observed that TRPV1 immunolabeling largely overlapped with CGRP in FG-positive neurons, suggesting that nerve fibers in gingival tissue express both TRPV1 and CGRP (Fig. 3a). Next, we tested whether the activation of TRPV1 in gingival tissue induces CGRP secretion. Oral administration of TRPV1 agonist, capsaicin, significantly increased CGRP production by TG explants when compared with the normal diet group (Fig. 3b). *Trpv1*^−/−^ mice treated with dietary capsaicin did not show any differences in CGRP production compared with the normal diet group, suggesting that the capsaicin-mediated effects observed in WT mice were TRPV1-dependent. Collectively, these results suggest that nerve fibers that innervate gingival tissues release CGRP upon TRPV1 activation.

### CGRP treatment suppresses LPS induced-osteoclastogenesis *in vitro*

**Figure 4:**
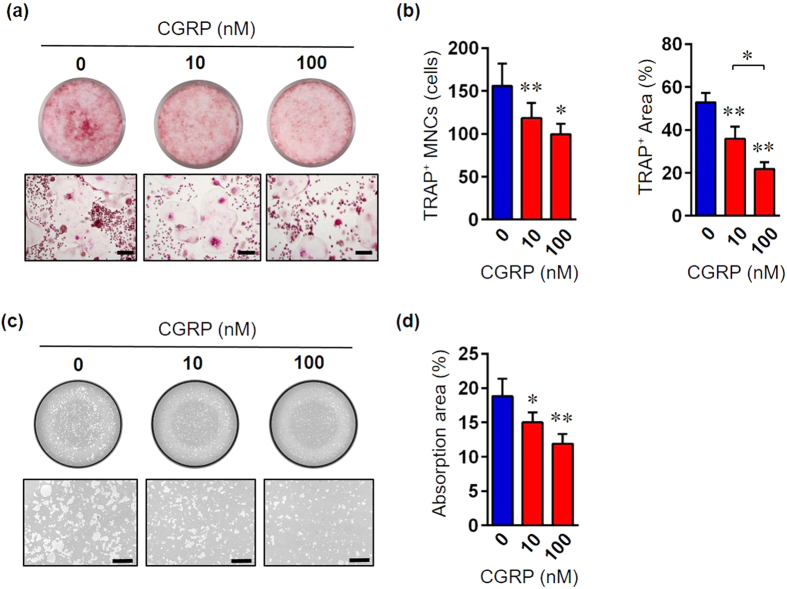
CGRP inhibits LPS induced-osteoclast differentiation *in vitro*. (**a**) Raw 264.7 cells were treated with RANKL (100 ng/mL)/*P. gingivalis* LPS (1 μg/mL) and cultured for 5 days with the indicated concentrations of recombinant CGRP. Representative images of TRAP staining at low magnification (upper panels) and high magnification (lower panels). Scale bars represent 500 μm. (**b**) The numbers (left) and the areas (right) of TRAP-positive multinucleated (≥3 nuclei per cells) cells (MNCs) (n = 5 in each group). All data are mean ± SD (*p < 0.05 and **p < 0.01 versus 0 nM CGRP or as indicated, by ANOVA). (**c**) Low magnification images (upper panels) and high magnification images (lower panels) of a bone resorption pit assay. Scale bars represent 400 μm.(**d**) Percentage of bone surface area resorbed (n = 5 in each group). All data are mean ± SD (*p < 0.05 and **p < 0.01 versus 0 nM CGRP, by ANOVA).

Alveolar bone destruction, a hallmark of periodontitis progression, is caused by imbalanced bone remodeling i.e. increased osteoclast and reduced osteoblast function. It has been reported that CGRP plays an important role in the modulation of osteogenesis under inflammatory conditions^19^. Therefore, we performed *in vitro* assays using osteoclast-like and osteoblast-like cells to explore the involvement of CGRP in osteogenesis. Under LPS-induced inflammatory conditions, pretreatment with recombinant CGRP significantly decreased the formation of tartrate-resistant acid phosphatase (TRAP)-positive osteoclast-like cells in a dose-dependent manner (Fig. 4a,b). In addition, the bone-resorbing activity of osteoclasts was significantly inhibited by CGRP pretreatment (Fig. 4c,d). However, CGRP treatment had no effect on osteoblastogenesis (Supplementary Fig. S2a–c). Altogether, these results suggest that CGRP inhibits LPS induced-osteoclast differentiation and subsequent bone resorption *in vitro*.

### TRPV1 activation by dietary capsaicin prevents periodontal bone destruction

**Figure 5:**
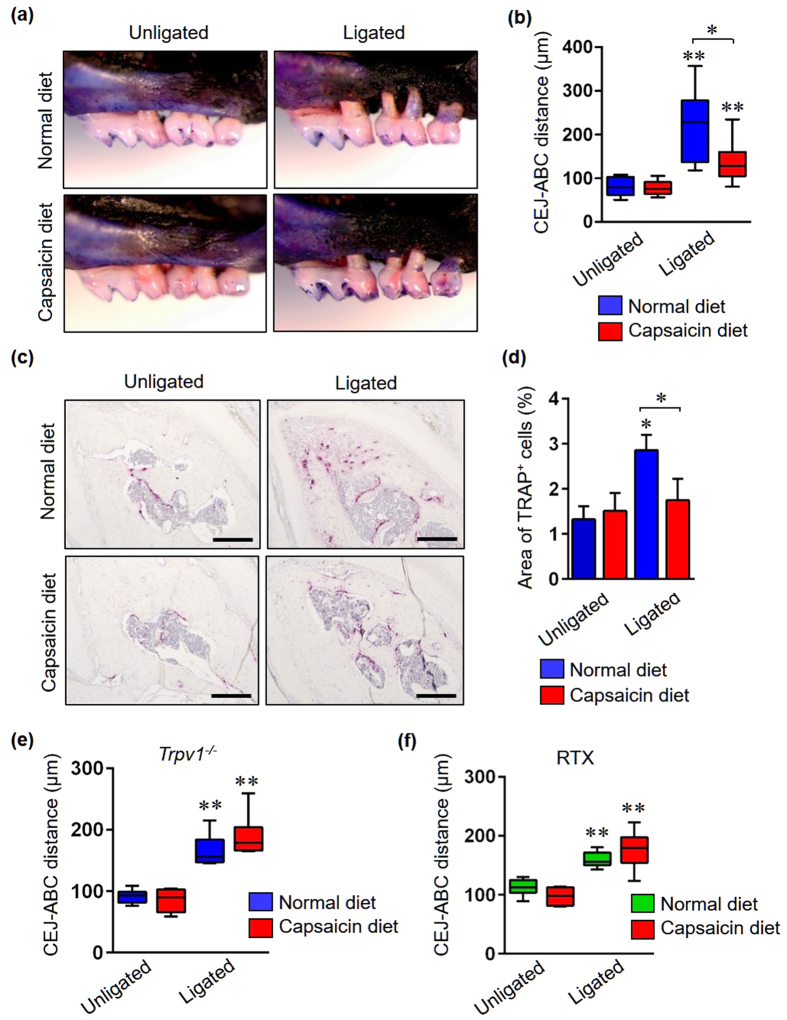
TRPV1 activation by dietary capsaicin prevents periodontal bone resorption. (**a**) Daily administration of capsaicin suppressed alveolar bone resorption in ligated mice. The normal diet group was fed standard laboratory chow, the other group was fed standard laboratory chow plus 0.01% (100 ppm) capsaicin for 14 days. On day 7, half of the mice from each group underwent ligation, and all mice were sacrificed for analysis on day 14. (**b**) The CEJ–ABC distance was significantly lower in ligated-mice fed with capsaicin compared to mice fed with the normal diet (n = 6 in each group) (**c**) Representative TRAP staining images in interradicular septa. Scale bars represent 200 μm. (**d**) Measurement of TRAP-positive areas adjacent to the bone surface in interradicular septa regions (n = 3 in each group). All data are mean ± SD (*p < 0.05 and **p < 0.01 versus unligated mice with normal diet or as indicated, by ANOVA). (**e**) Quantification of alveolar bone loss in *Trpv1*^−/−^ and (**f**) RTX-treated mice (n = 6 in each group). All data are mean ± SD (**p < 0.01 versus unligated mice with normal diet, by ANOVA).

Based on the above findings, we hypothesized that activation of neuronal TRPV1 in gingival tissues might protect against alveolar bone resorption in periodontal disease. As predicted, administration of the dietary TRPV1 agonist capsaicin, significantly suppressed bone destruction in the ligated group (Fig. 5a,b and Supplementary Fig. S3a). There were no differences in mean body weight or microbial abundance between the capsaicin-diet group and the normal diet group (Supplementary Fig. S3b,c). Additionally, the portion of TRAP positive cells adjacent to the bone surface in interradicular septa was significantly decreased in capsaicin-treated mice with ligation compared to the normal diet-treated mice with ligation (Fig. 5c,d). Capsaicin treatment did not rescue the bone loss in neither ligated-*Trpv1*^−/−^ mice nor ligated-RTX mice (Fig. 5e,f and Supplementary Fig. S4a,b), suggesting that neuronal TRPV1 signaling in gingival tissues prevents periodontal bone destruction.

### CGRP-immunopositive nerve fibers are in close vicinity of osteoclasts at the alveolar bone surface

**Figure 6:**
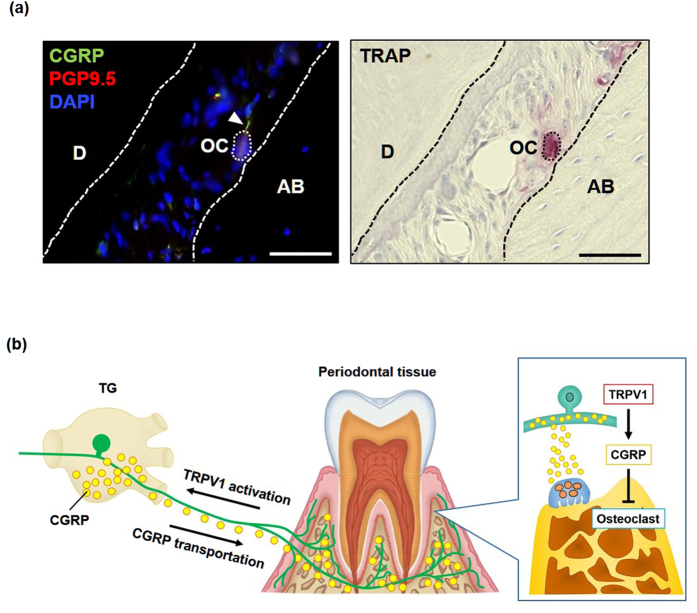
Distribution of CGRP-immunopositive nerve fibers in gingival tissue. (**a**) Immunofluorescent staining for CGRP and PGP9.5, a neuronal maker (left panel) and TRAP staining (right panel) in consecutive tissue sections of gingival tissues. The arrowhead indicates CGRP-containing nerve fibers that are in the vicinity of osteoclasts on the alveolar bone surface. OC, osteoclasts. AB, alveolar bone. D, dentin. Scale bars represent 50 μm. (**b**) Model of the relative roles of TRPV1 and CGRP on bone remodeling in periodontitis. Activation of TRPV1 on sensory neurons innervating gingival tissues induces afferent input to TG, which results in the synthesis of neuropeptides such as CGRP. Anterograde axonal transportation of CGRP to peripheral tissue and subsequent release of CGRP leads to the inhibition of osteoclast differentiation in alveolar bone.

To evaluate the role of CGRP-immunoreactive nerve fibers in osteogenesis, we performed immunostaining of gingival tissue. We found that CGRP-immunopositive nerve fibers are in close contact with TRAP-positive osteoclasts (Fig. 6a), suggesting that they may affect osteogenesis by directly releasing CGRP in the gingival microenvironment. Collectively, these results led us to propose a working model in which the activation of TRPV1-expressing sensory neurons in gingiva induces local CGRP secretion, which in turn suppresses osteoclastogenesis under inflammatory conditions as occurring in periodontitis (Fig. 6b).

## Discussion

In this study we demonstrated that TRPV1 activation in gingival nerves evokes the local release of neuropeptide CGRP, which suppresses alveolar bone loss in the ligature-induced periodontitis murine model. Furthermore, our *in vitro* studies revealed that CGRP signaling inhibited the osteoclastogenesis induced by microbial products derived from periodontopathic bacteria. Our work supports the notion that the peripheral nervous system contributes to the pathophysiology of periodontal diseases via neuropeptide secretion.

Periodontitis is a chronic infectious inflammatory disease that damages the periodontal soft tissue and gradually destroys the tooth-supporting alveolar bone^34^. Öztürk *et al*. have reported the presence of TRPV1 in gingival biopsies and that its expression was down-regulated in samples obtained from patients with periodontitis compared with healthy controls^35^. CGRP has been detected in human gingival tissue^36^, and the amount of CGRP secreted in GCF differed between subjects with or without periodontitis^27^,^37^,^38^. Immunostainings have shown the co-localization of TRPV1 and CGRP in peripheral nerve fibers in gingival tissues that project from TG^39^, and the activation of TRPV1 evokes CGRP release from rat buccal mucosa of trigeminal field of innervation^40^. Our study identified a functional link between TRPV1 and CGRP, and demonstrated their pathophysiological interaction in an experimental periodontitis model.

*Porphyromonas gingivalis (P. gingivalis*) is a Gram-negative organism that is considered to be the most important agent in the initial process of periodontitis^41^. It is interesting to note that the TRPV1 agonist, capsaicin, exhibited antimicrobial activity against the growth and biofilm formation of *P. gingivalis*^42^. Anti-inflammatory properties of capsaicin have been confirmed both *in vitro*^43^ and in animal studies^44^. Furthermore, the administration of capsaicin increases the secretion of saliva, which possesses many important functions including antimicrobial activity, mechanical cleaning action, and lubrication of the oral cavity^45^. Ishimaru *et al*. demonstrated that specific expression of TRPV1 in taste buds of the tongue improved salty taste detection^46^. Altogether, these reports suggest that capsaicin has beneficial effects on various oral diseases and malfunctions such as periodontitis, dry mouth syndrome, and taste disorders.

In this study we identified a regulatory role for CGRP in osteoclastogenesis by highlighting the inhibitory effects of CGRP in the formation of TRAP positive multinucleated cells. These findings are consistent with previous studies showing that CGRP signaling has marked effects on bone metabolism. First, osteoclasts express functional receptors for CGRP^47^. Second, CGRP-immunoreactive nerve fibers are widely distributed in bone tissue such as periosteum and bone marrow^48^, and an increase in CGRP-immunoreactive nerve fibers was demonstrated in the repair process of experimental bone defects^49^ as well as in bone remodeling of orthodontic tooth movement^50^. Third, CGRP-deficient mice show osteopenia and accelerated bone loss with aging^51^,^52^. Finally, the administration of CGRP has anti-osteoclastogenic effects both *in vitro*^19^ and *in vivo*^53^. Collectively, and in line with our findings, these reports suggest that CGRP signaling in pre-osteoclasts can inhibit their differentiation into osteoclasts and prevent subsequent alveolar bone loss.

In osteoclast lineages, the sequential activation of NF-κB in progenitor cells is essential for RANKL-induced osteoclast differentiation, activity, and survival^54^. Dion *et al*. observed that CGRP modulates immune cell function by inhibition of phospho-IKKβ, which prevents IκBα degradation and activation of NF-κB^55^. Wang *et al*. reported that CGRP suppresses osteoclastogenesis and bone resorption by inhibiting RANKL-induced NF-κB activation in osteoclast precursors^19^. Collectively, the inhibition of osteoclastogenesis by CGRP in our model may be attributable to inhibitory effects on RANKL-induced activation of NF-κB in osteoclast precursors. Elucidating the intracellular signaling pathways via neuropeptide receptors may shed light on neuro-osteogenic crosstalk.

While our data suggest a protective role of TRPV1 activation against ligature-induced bone loss, the role of TRPV1 in bone metabolism remains controversial. In accordance with our findings, Kobayashi *et al*. reported that TRPV1 signaling suppressed bone resorption by osteoclasts and attenuated inflammatory bone loss induced by the administration of LPS *in vivo*^56^. In the experimental osteoarthritis rat model, pathological changes in the joint (bone erosion, trabecular damage) were significantly reduced by pre-treatment with capsaicin^13^. Conversely, Rossi *et al*. reported that genetic deletion or pharmacological inhibition of TRPV1 reduced osteoclast activity *in vitro* and prevented ovariectomy-induced bone loss *in vivo*^12^. Consistently, pharmacological blockade of TRPV1 by capsazepine protected mice against overiectomy-induced bone loss by suppressing osteoclast formation^57^. These reported discrepancies of TRPV1 agonists vs. antagonists on bone metabolism might be due to differences in the type of disease model, as well as differences in the doses of administrated compounds. It has been reported that TRPV1 agonists can induce biphasic, dose-dependent effects on the vasculature^58^. Overactivation of TRPV1 upon administration of high doses of its agonist could induce channel desensitization and, hence, similar to treatment with an antagonist, may result in opposite effects. In addition, recent evidence suggests that aging reverses the role of TRPV1 channel from anti-inflammatory to pro-inflammatory^59^. This functional switch of TRPV1 signaling in aging could also be a potential explanation for the discrepancies of its effects on bone metabolism.

One limitation of our study is the lack of CGRP detection in gingival tissue *in vivo* after dietary capsaicin treatment. This is most likely because the concentration of CGRP in gingival tissues was below the detection limit of ELISA. This might be due to the rapid degradation of CGRP, but not other neuropeptides, by components of the GCF^37^. Alternatively, we compared the concentration of CGRP released from isolated TG with neuropeptides synthesized prior to axonal transport to the periphery. Hence, the increased CGRP secretion in TG induced by dietary administration of capsaicin reached higher amounts than local CGRP release in gingival tissue.

In conclusion, we identified that periodontal alveolar bone resorption is associated with the activation of TRPV1^+^ peripheral neurons and with the regulatory effects of the neuropeptide CGRP on osteoclastogenesis. Our study suggests that the TRPV1 channel could be a potential therapeutic target for diseases associated with inflammatory bone resorption, including periodontitis.

## Methods

### Reagents and antibodies

Capsaicin (≥95% purity, from *Capsicum sp.*) was purchased from Sigma-Aldrich Corporation (St. Louis, MO, USA). RTX and recombinant CGRP were obtained from LC laboratories (Woburn, MA, USA) and Phoenix pharmaceuticals, Inc. (Burlingame, CA, USA), respectively. *P. gingivalis* LPS was purchased from InvivoGen (San Diego, CA, USA).

### Mice

All experiments were performed in accordance with the Regulations and Guidelines on Scientific and Ethical Care and Use of Laboratory Animals of the Science Council of Japan, enforced on June 1, 2006, and approved by the Institutional Animal Care and Use Committee at Niigata University (permit number 112–5). Six- to 8-week-old male C57BL/6 mice were purchased from Japan SLC, Inc. (Shizuoka, Japan). TRPV1-deficient mice^6^ were kindly provided by Dr. Makoto Tominaga (National Institutes of Natural Sciences, Okazaki, Japan). For neonatal ablation of TRPV1^+^ sensory neurons, C57BL/6 mice were treated with RTX (50 μg/kg s.c.) on days 1, 2, and 7 after birth^31^. Validation of TRPV1^+^ neuronal ablation was performed by eye-wiping test. Briefly, a drop of 0.01% (w/v) capsaicin in saline was put onto the cornea of the eye and the number of defensive wiping movements was counted for 1 min. The loss of eye wipe response indicates inactivation of TRPV1 in afferent neurons and only mice that showed a significant decline in the counts were used in the experiments. All mice were acclimatized under specific pathogen-free conditions and fed regular chow and sterile water throughout the experiment.

### Polymerase chain reaction (PCR) and gel electrophoresis

Total RNA was isolated from tissues and cells using TRI Reagent^®^ (Molecular Research Center, Inc., Cincinnati, OH, USA). cDNA was synthesized using Transcriptor Universal cDNA Master (Roche Molecular Systems, Inc., Branchburg, NJ, USA). Conventional PCR was performed in a 20 μL reaction volume using GoTaq polymerase (Promega Corporation, Madison, WI, USA) with the following protocol: predenaturation at 94 °C for 5 min followed by 30 cycles of denaturation at 94 °C for 15 s, annealing at 60 °C for 15 s, extension at 72 °C for 30 s, and a final extension step at 72 °C for 10 min by using a GeneAmp^®^ PCR System 7700 (Applied Biosystems, Carlsbad, CA, USA). PCR products were run on 1.5% agarose gels and visualized using SYBR^®^ Safe DNA (Invitrogen Corporation, Carlsbad, CA, USA). *Glyceraldehyde-3-phosphate dehydrogenase (Gapdh*) was amplified using forward primer 5′-TCAACAGCAACTCCCACTCTT-3′ and reverse primer 5′-ACCCTGTTGCTGTAGCCGTAT-3′. *Trpv1* was amplified using forward primer 5′-AGCTGCAGCGAGCCATCACCA-3′and reverse primer 5′-ATCCTTGCCGTCCGGCGTGA -3′.

### Ligature-induced periodontitis model

To induce bone loss, a 5–0 silk ligature was tied around the maxillary second molar without damaging the nearby gingiva under anesthesia. The ligatures remained in place in all mice throughout the experimental period. After a 7-day ligature, the following assessments were performed. Unligated mice that received anesthesia only were used as controls.

### Measurement of alveolar bone loss

Following defleshing, the bones were subjected to brushing and bleaching. The maxillae were stained with 1% methylene blue to delineate the CEJ and ABC. The distance from the CEJ to ABC of the mesial roots of the maxillary second molar was measured on images obtained using a stereomicroscope (DP2-BSW; OLYMPUS, Tokyo, Japan). The measurement of alveolar bone loss in mice was performed in a blind manner.

### Retrograde tracing of gingival neurons

The mice were anaesthetized and positioned with the mouth held open for observation under a surgical microscope. A 4% solution of retrograde nerve tracer Fluoro-Gold™ (Abcam, Burlingame, CA, USA) was carefully injected into the palatinal and buccal gingiva of maxillary molars using a Hamilton microsyringe (5 μL each part). After 24 hours, the TG were dissected and FG fluorescence was observed under fluorescence microscopy (Biozero BZ-8000; Keyence Corporation, Osaka, Japan).

### Immunostaining

TG were fixed in 4% paraformaldehyde for 24 h and embedded in paraffin. Tissue sections on slides were deparaffinized and incubated with anti-TRPV1 antibody (Alomone Labs, Jerusalem, Israel) and anti-CGRP antibody (Abcam) at 4 °C overnight. The slides were then incubated with Alexa Fluor 594 -conjugated anti-rabbit secondary antibody (Abcam) for TRPV1 and Alexa Fluor 488-conjugated anti-goat secondary antibody (Abcam) for CGRP at room temperature for 1 h. For histological analysis of alveolar bone tissues, decalcified and embedded sections were double-stained by anti-CGRP antibody and anti-PGP9.5 antibody (Novus Biologicals, Littleton, CO, USA) with appropriate Alexa Fluor-conjugated secondary antibodies. Slides were mounted with VECTASHIELD^®^ HardSet™ Mounting Medium with DAPI (Vector laboratories, Burlingame, CA, USA) and analyzed by fluorescence microscopy (Keyence Corporation).

### Measurement of CGRP

Dissected TG were weighed and placed in Dulbecco’s Modified Eagle Medium: Nutrient Mixture F-12 (DMEM/F-12) (Gibco, Grand Island, NY, USA) medium containing 10% Fetal Bovine Serum (FBS) (Gibco), 100 IU/ml penicillin and 100 mg/ml streptomycin (Gibco) at 37 °C in 5% CO_2_. After incubation for 24 h, supernatant was collected and CGRP concentration was measured using a commercially available ELISA kit according to the manufacturer’s instructions (Cayman, Ann Arbor, MI, USA). Optical densities were measured using a microplate spectrometer (Model 680; Bio-Rad Laboratories, Hercules, CA, USA). Data were normalized to the weight of each collected tissue.

### Determination of osteoclastogenesis

Osteoclast precursor cells, Raw 264.7 cells were maintained in Minimum Essential Medium (MEM) α (Gibco) supplemented with 10% FBS, 100 IU/mL penicillin, and 100 mg/mL streptomycin at 37 °C in 5% CO_2_ in humidified air. To induce differentiation of RAW264.7 cells, they were treated with mouse RANKL (100 ng/mL) (eBioscience, San Diego, CA, USA) and *P. gingivalis* LPS (1 μg/mL) in the presence or absence of CGRP (10, 100 nM) for 7 days. The culture medium was replaced every 3 days. For morphological analysis, the cells were stained using a TRAP staining kit (Primary Cell Co., Ltd., Sapporo, Japan). The numbers of multinucleated TRAP-positive osteoclasts per well in a 96 well plate were counted under microscopic examination. For semi-quantitative evaluation, the area of TRAP-stained cell surface was measured using Image J software (National Institutes of Health, Bethesda, MD, USA) and normalized to the surface area of wells in a 96 well plate. A bone resorption pit assay was conducted using a 96-well Corning Osteo Assay Surface plate (Corning, Inc., Corning, NY, USA) following the manufacturer’s instructions. The percentage of resorption area on well surface was quantified using Image J software.

### Osteoblast differentiation and mineralization

MC3T3-E1 cells, an osteoblastic cell line, were grown in RPMI medium (Sigma) supplemented with 10% FBS, 100 IU/mL penicillin and 100 mg/mL streptomycin at 37 °C in 5% CO_2_ in humidified air. Osteoblast differentiation was induced by Osteoblast-Inducer Reagent (Takara Bio Inc., Shiga, Japan), a cocktail of L-ascorbic acid, dexamethasone and β-glycerophosphoric acid in the presence or absence of CGRP (10, 100 nM) for 10 days. The culture medium was replaced every 3 days. Alkaline phosphatase (ALP) activity was determined by the TRACP & ALP double-stain kit (Takara Bio Inc.) according to the manufacturer’s protocol. Results were normalized to total cellular protein contents. The protein concentration of the lysates was determined using Pierce™ BCA Protein Assay Kit (Thermo Scientific, Rockford, IF, USA). Mineralization was determined using Alizarin red staining. The cells were fixed with 4% PFA and stained with Alizarin red to detect calcification. For quantification, cells were lysed and the absorbance was read with a microplate spectrometer (Bio-Rad Laboratories).

### Determination of bacterial accumulation

A sterile paper-point (Zipperer Absorbent Paper Points, VDW GmbH, Munich, Germany) was held against the gum line for 5 s in the oral cavity and then vortexed thoroughly in 1 ml of saline. Bacterial DNA was extracted from these samples using a QIAampDNA Blood Mini Kit (Qiagen, Hilden, Germany). Quantitative real-time PCR was performed with 10 ng of sample DNA in a final volume of 20 μL per reaction using Fast Start Essential DNA Green Master (Roche) on a LightCycler^®^ 96 System (Roche). The universal 16 s rRNA sequence was amplified with a predenaturation at 95 °C for 30 s, followed by 40 cycles of 95 °C for 10 s and 60 °C for 30 s using a specific primer for universal 16S rRNA; forward primer 5′-ACTCCTACGGGAGGCAGCAGT-3′ and reverse primer 5′-ATTACCGCGGCTGCTGGC-3′. The Ct values obtained in the PCR were converted to gene copy numbers to estimate the abundance of bacterial genomes.

### TRAP staining of paraffin-embedded tissue samples

The fixed maxillae of mice from each group were dissected, decalcified, and embedded. Serial sections were obtained in the sagittal direction along the long axis of the teeth. The sections were stained using a TRAP staining kit and counterstained for hematoxylin. The area of TRAP positive cell surface in an interradicular septa region was measured using ImageJ software and was normalized to bone surface in the region.

### Statistical analysis

All experiments were independently repeated at least twice, on separate days. All data are expressed as the mean ± standard deviation (SD). Statistical analyses were performed using GraphPad Prism (GraphPad Software, Inc., San Diego, CA, USA). The Mann-Whitney U test was applied for two-group comparisons, and the ANOVA was used for multiple-group comparisons. A p-value of <0.05 was considered statistically significant.

## Additional Information

**How to cite this article**: Takahashi, N. *et al*. Neuronal TRPV1 activation regulates alveolar bone resorption by suppressing osteoclastogenesis via CGRP. *Sci. Rep.* **6**, 29294; doi: 10.1038/srep29294 (2016).

## Supplementary Material

Supplementary Information
