# Supplementary material for: Neuronal TRPV1 activation regulates alveolar bone resorption by suppressing osteoclastogenesis via CGRP
Source: Sci Rep. 2016 Jul 8;6:29294. doi: 10.1038/srep29294 (PMC4937344; doi:10.1038/srep29294)
Supplement: Supplementary Information [file srep29294-s1.pdf]

Supplemental Figures

Neuronal TRPV1 activation regulates alveolar bone resorption by suppressing osteoclastogenesis via CGRP.

Naoki Takahashi[1,2,3], Yumi Matsuda[1,2], Keisuke Sato [1,2], Petrus R. de Jong[4], Samuel Bertin[5], Koichi Tabeta[2] and Kazuhisa Yamazaki[1]*

Affiliations

[1]Laboratory of Periodontology and Immunology, Division of Oral Science for Health Promotion, Niigata University Graduate School of Medical and Dental Sciences, Niigata, Japan.

[2]Division of Periodontology, Department of Oral Biological Science, Niigata University Faculty of Dentistry, Niigata, Japan.

[3]Research Center for Advanced Oral Science, Niigata University Graduate School of Medical and Dental Sciences, Niigata, Japan.

[4]Sanford Burnham Prebys Medical Discovery Institute, NCI-Designated Cancer Center, La Jolla, CA, USA.

[5]Department of Medicine, University of California, San Diego, La Jolla, CA, USA.

*Correspondence: kaz@dent.niigata-u.ac.jp

Supplemental Figure S1

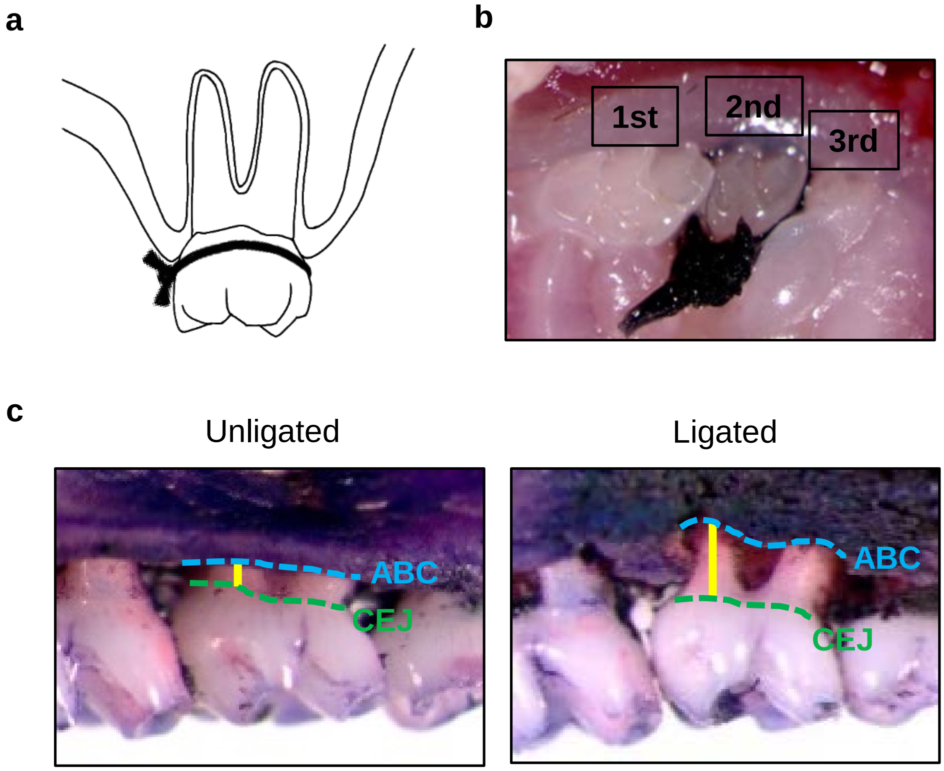

Ligature-induced periodontitis murine model.
(a) Scheme of ligature around the cervical portion of second molar in the experimental periodontitis model. (b) Photograph of the ligature around the 2nd molar at day 7 after ligation. (c) Representative stereoscope images in unligated and ligated groups at day 7. Blue dot line and green dot line represent ABC and CEJ, respectively. The amount of alveolar bone loss was indicated by the solid yellow line.

Supplemental Figure S2.

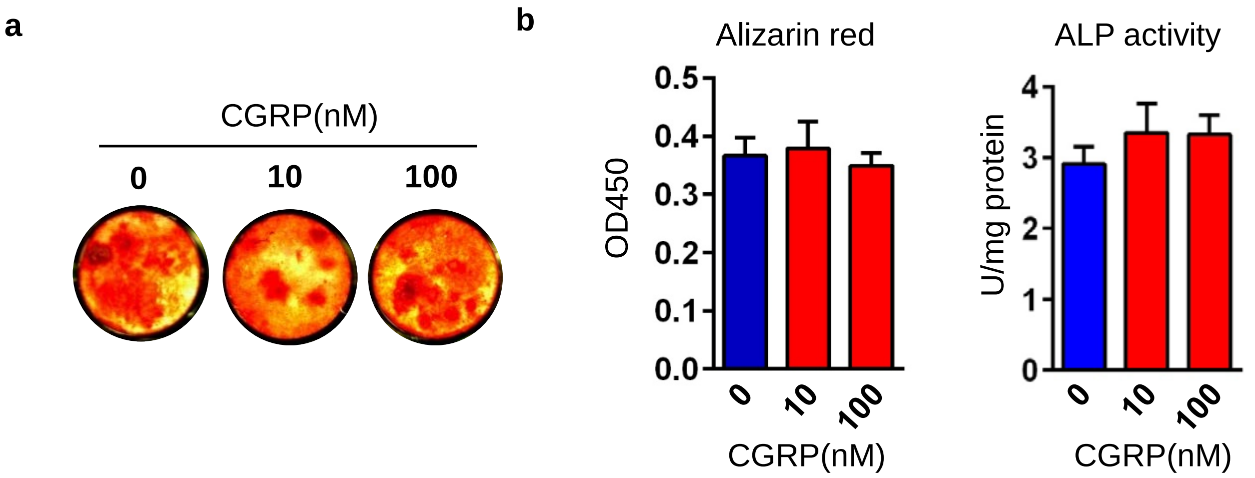

Effect of CGRP on osteoblast differentiation *in vitro*.

(a) Alizarin Red staining of mineralized nodules in the osteoblastic cell line, MC3T3-E1 cells differentiated by adding Osteoblast-Inducer Reagent into culture medium in the presence of indicated concentrations of CGRP for 10 days. (b) Quantification of osteoblastic activities using Alizarin Red staining (left) and ALP measurement (right) (n=5 in each group). All data are mean ± SD.

Supplemental Figure S3.

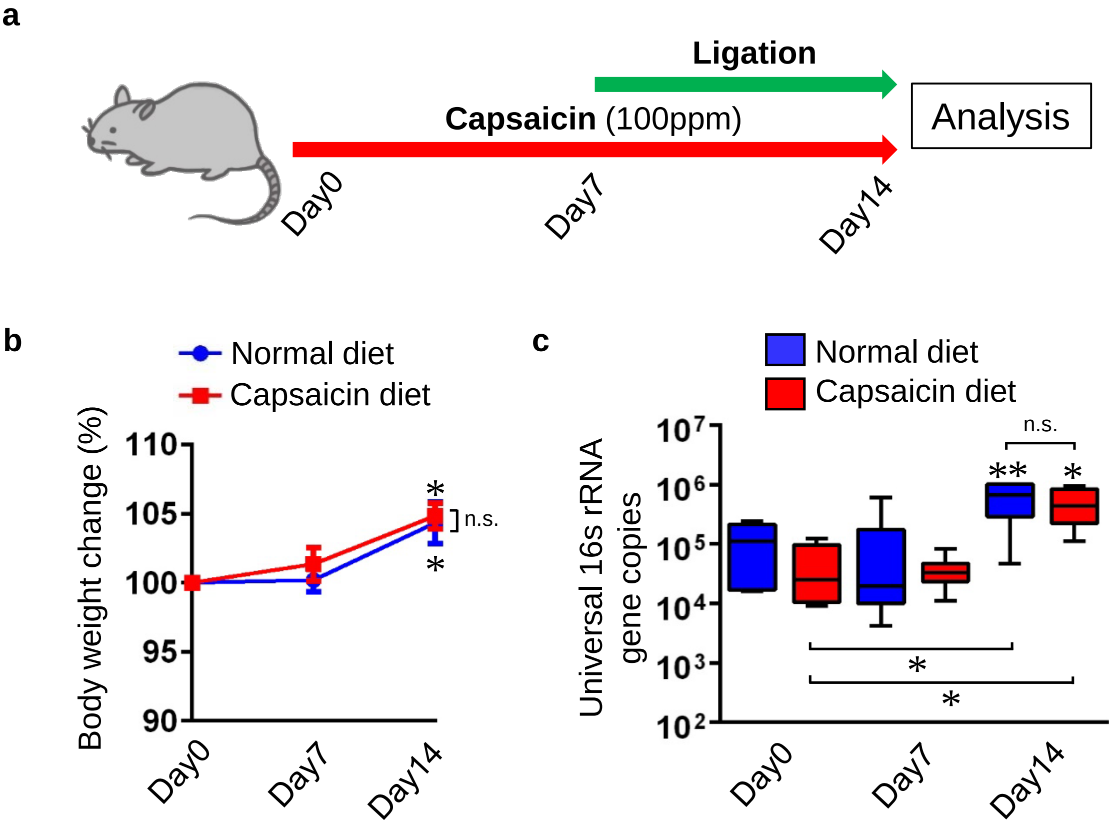

Effect of dietary capsaicin treatment in the ligature-induced periodontitis model.
(a) Experimental design of capsaicin treatments in the mice model. (b) Mean body weights of mice fed normal chow or capsaicin diet were monitored on Day 0, 7 and 14. (c) Quantification of bacterial abundance was performed by PCR using universal 16s rRNA primers at the indicated time points. Subgingival plaque samples were obtained from each mouse by placing sterile paper points (n=6 in each group). All data are mean ± SD. (*$p<0.05$ and **$p<0.01$ versus normal diet on Day0 or as indicated, n.s.; not significant, by ANOVA).

Supplemental Figure S4.

a

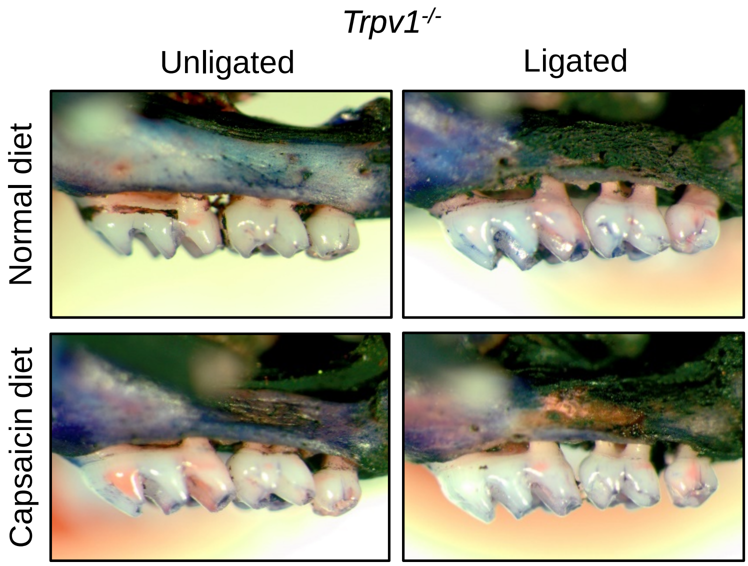

b

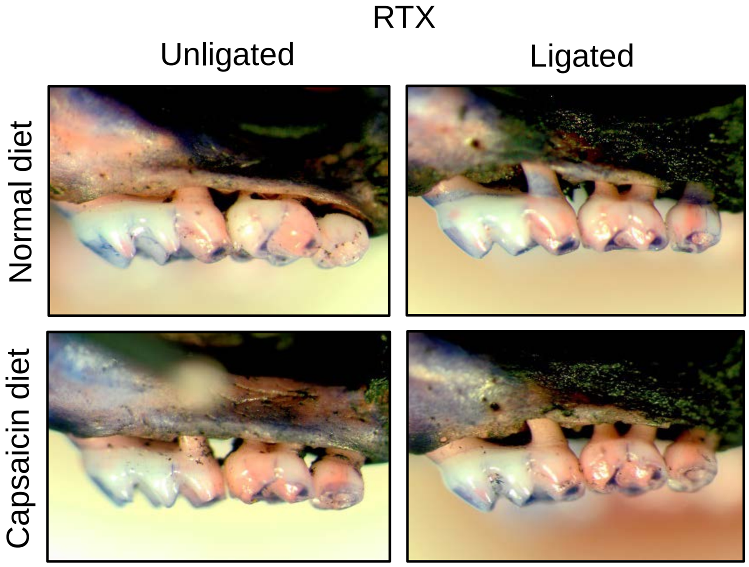

No protective effects of capsaicin against bone destruction in *Trpv1$^{-/-}$* and RTX-treated mice. Representative stereoscope photos of defleshed maxilla and measurement of bone loss from *Trpv1$^{-/-}$* (a) and RTX-treated mice (b).
